# ﻿*Tiliasaxatilis* (Malvaceae), a new species from limestone areas of Guangxi, China

**DOI:** 10.3897/phytokeys.251.141836

**Published:** 2025-01-29

**Authors:** Zhao-Cen Lu, Shi-Li Chang, Ming-Lin Mo, You-Dong Wu, Wei-Bin Xu

**Affiliations:** 1 Guangxi Key Laboratory of Plant Conservation and Restoration Ecology in Karst Terrain/Nonggang Karst Ecosystem Observation and Research Station of Guangxi, Guangxi Institute of Botany, Guangxi Zhuang Autonomous Region and Chinese Academy of Sciences, Guilin, 541006, Guangxi, China Guangxi Institute of Botany, Guangxi Zhuang Autonomous Region and Chinese Academy of Sciences Guilin China; 2 College of Life Sciences, Guangxi Normal University, Guilin, 541006, Guangxi, China Guangxi Normal University Guilin China

**Keywords:** Malvaceae, morphology, new species, taxonomy, *Tiliatuan*

## Abstract

*Tiliasaxatilis* Z.C.Lu & W.B.Xu, a new species was discovered in limestone areas of Guangxi, China. The morphology shows that *T.saxatilis* is similar to *T.tuan* Szyszyl., but differs by having leaf blades that are oblong or ovate-oblong, entire margins, fruit ellipsoid, 5-angled, apex acute.

## ﻿Introduction

The genus *Tilia* L. with 23–40 species in the family Malvaceae is distributed in Europe, North America and East Asia where in occurs primarily in temperate and subtropical regions (Zhuge and Tang 1995; Tang and Zhuge 1996; Pigott 2002; Tang et al. 2007; Pigott 2012). *Tilia* is easily recognised by its unique tongue-like bracts adnate to the peduncle of the inflorescence and leaf blades base oblique (Chang 1989; Tang et al. 2007).

The genus *Tilia* was firstly described by Linnaeus (1753) in the "Species Plantarum" and the earlier taxonomic treatments mainly focused on European and American species. Chang (1989) presented a comprehensive treatment of Chinese *Tilia* in the "Flora Republicae Popularis Sinicae", which included a key and descriptions of 32 species. Pigott (2012) provided a very comprehensive taxonomic treatment of *Tilia* in his monograph, accepted 23 species, of which two species were in North American, four species ranging from Europe to West Asia and 17 species in East Asia and also confirmed China is the centre of species diversity for the genus *Tilia*, with 15 species (11 endemic).

During the investigation of plant diversity in the assessment area of Southwest Karst National Park (which is currently being prepared) in July, September and October 2023, we collected a species of *Tilia* with flowers and mature fruits from limestone forests in Dahua County and Du’an County, Hechi City, Guangxi, China. After carefully checking the morphological characters of the specimens, comprehensively consulting relevant literature (Chang 1989; Zhuge and Tang 1995; Tang and Zhuge 1996; Pigott 2002; Tang et al. 2007; Pigott 2012), consulting herbarium specimens and examining the other related species of *Tilia*, we confirmed it is a new species. It is described below.

## ﻿Materials and methods

Specimens of this new species were collected from Dahua County and Du’an County, Hechi City, Guangxi, China. After that, we carefully studied relevant literature and the morphological characters of the specimens, which involved measuring and recording the size, shape and colour of bark, branchlets, winter buds, leaves, cymes, bracts, pedicel, sepals, petals, stamens, ovary, fruit, seeds and so on. We examined herbarium specimens at BJFC, IBK, IBSC, IFP, HIB, KUN, PE and WUK (Herbarium codes follow Thiers 2024). The other related species of *Tilia* were examined in online images from the Chinese Virtual Herbarium (https://www.cvh.ac.cn/), JSTOR Global Plants (https://plants.jstor.org/) and Kew Herbarium Catalogue (http://apps.kew.org/herbcat/gotoHomePage.do). The morphological description for the new species is based on the cited type specimens (holotype, isotypes and paratypes) below and general terminology follows the "Flora of China" (Tang et al. 2007).

## ﻿Taxonomic treatment

### ﻿ Tilia saxatilis

Taxon classificationPlantaeMalvalesMalvaceae

Z.C.Lu & W.B.Xu sp. nov.

F6CAD151-C840-54CF-8014-305006360C57

urn:lsid:ipni.org:names:77355914-1

**Figure 1.**
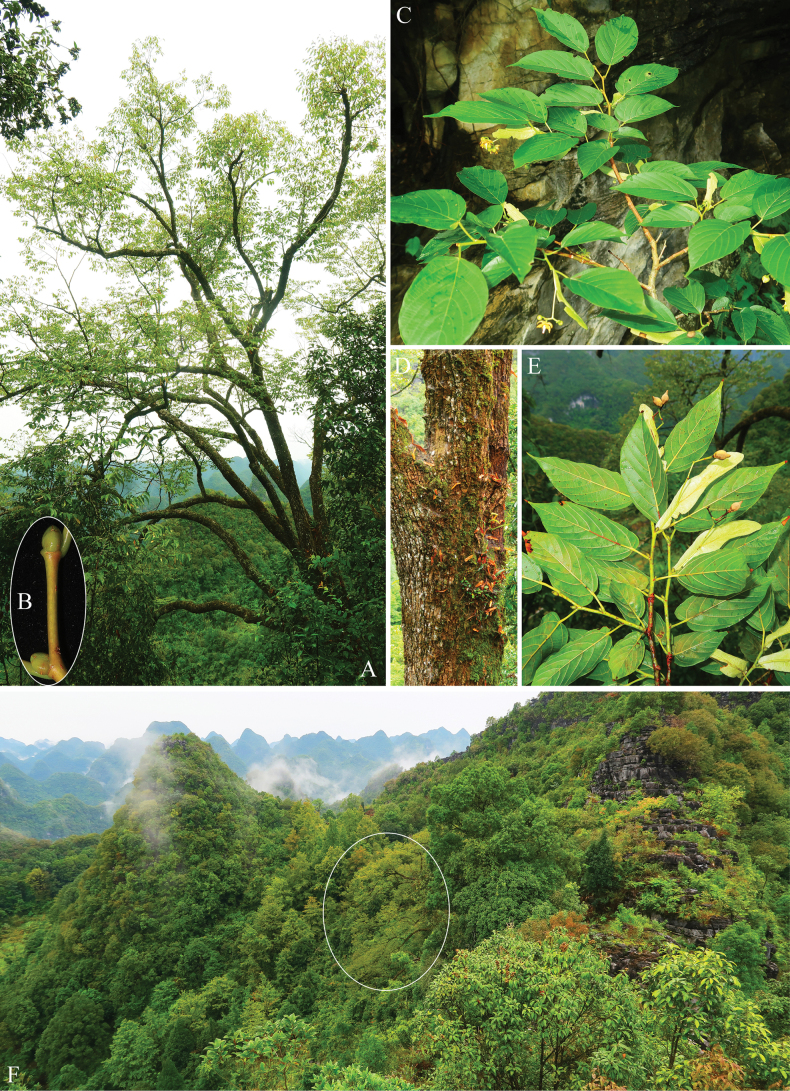
*Tiliasaxatilis* sp. nov. **A** habit **B** winter buds **C** flowering branches **D** trunk **E** fruiting branches **F** habitat (White circle shows where the new species grow).

**Figure 2.**
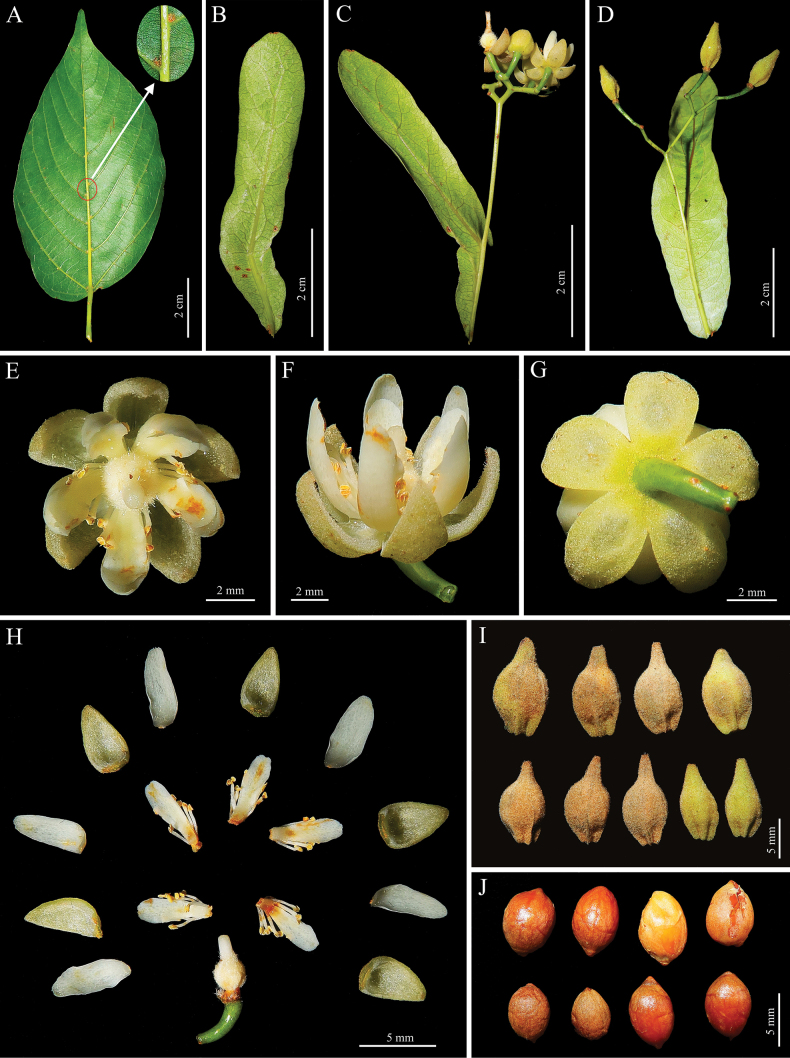
*Tiliasaxatilis* sp. nov. **A** leaf in abaxial view (Red circle shows tuft domatia in vein axils) **B** bract in abaxial view **C** bract and cyme **D** bract and infructescence **E** flower in frontal view **F** flower in lateral view **G** flower in dorsal view **H** dissection of flower **I** fruits **J** seeds.

**Figure 3.**
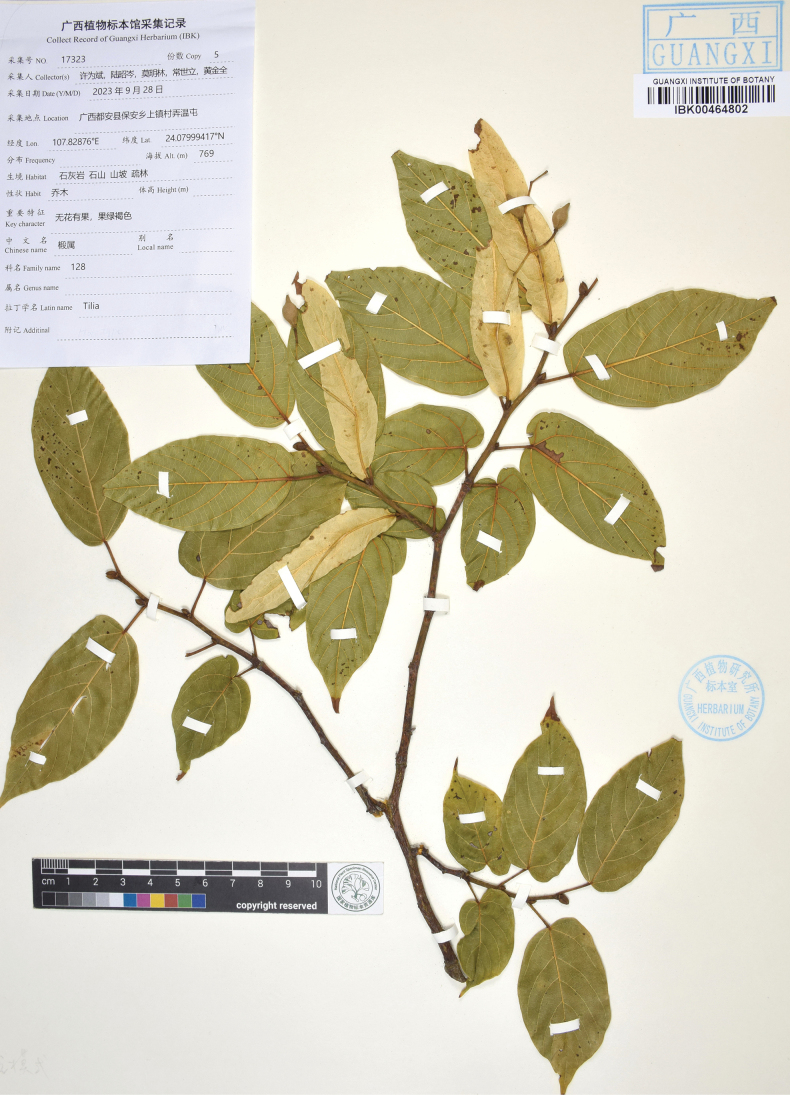
The holotype sheet of *Tiliasaxatilis* (IBK).

Figs 1 , 2 , 3 Chinese name: shí shān duàn (石山椴)

#### Diagnosis.

This new species is similar to *Tiliatuan* Szyszyl., but differs in having leaf blades that are oblong or ovate-oblong (vs. narrowly ovate or ovate-oblong to ovate-orbicular), margins entire (vs. entire or with a few minute teeth near apex or prominently dentate); fruit ellipsoid (vs. globose or obovoid-globose), 5-angled (vs. not ridged), apex acute (vs. rounded).

#### Type.

China • Guangxi: Hechi City, Du’an County, Bao’an Town, Shangzhen Village, Nongwen, around the point 24.07999417°N, 107.82876°E, limestone slope, alt. 769 m, 28 September 2023, *W. B. Xu, Z. C. Lu, M. L Mo, S. L. Chang & J. Q. Huang 17323* (holotype: IBK00464802; isotypes: IBK00464803, IBK00464804, IBSC, PE).

#### Description.

Trees 5–15 m tall, DBH 10–80 cm. Bark dark grey; branchlets glabrous; winter buds ovoid, glabrous or slightly hairy at tip. Petiole 0.8–2 cm long, glabrous; leaf blades oblong or ovate-oblong, (3.5–)4.5–10(–14.4) cm long, 2–5.5(–6.2) cm wide, thickly papery, glabrous on both sides, with brown tuft domatia in vein axils of abaxial surface, lateral veins 7–8 pairs, raised on abaxial surface, reticulate veins distinct abaxially, base oblique, truncate or cordate, margins entire, apex acuminate. Cymes 5–16-flowered, 3.5–6 cm long, peduncles glabrous. Bracts narrowly oblong, 3.8–9 cm long, 1–2 cm wide, adnate to peduncle for 1/3–2/5 of its length, glabrous or adaxially slightly hairy along mid-vein, apex obtuse, sessile. Pedicels 2–4 mm long, glabrous. Sepals 5, ovate, 4.5–5 mm long, abaxially tomentose, adaxially tomentose or with long tomentose at base. Petals oblong, 5–6 mm long, glabrous. Stamens ca. 2 mm long, glabrous; staminodes 5, slightly smaller than petals, glabrous. Ovary densely tomentose; style 1–2 mm long, glabrous. Fruit ellipsoid, 5-angled, 8–12 mm long, 5–6 mm wide, densely appressed tomentose, apex acute; exocarp woody, hard, indehiscent. Seed ellipsoid, ca. 5 mm long.

#### Etymology.

The specific epithet ‘*saxatilis*’ refers to the limestone habitats of this new species.

#### Phenology.

Flowering July and fruiting from September to October.

#### Distribution and habitat.

*Tiliasaxatilis* has only been collected from five localities restricted to central Guangxi of China. It grows sporadically in forests on limestone slopes, rare on peaks, at an elevation of 700–950 m. The type localities are typical limestone karst landform and belong to the southern subtropical monsoon climate areas, the average annual temperature being 18–21 °C; the annual sunshine duration is 1220–1590 hours and the annual accumulated temperature is about 6300 °C; the annual average relative humidity is 74–80%; the average annual rainfall is 1250–1680 mm and the evaporation is 1210–1650 mm.

#### Conservation status.

The new species has been found in two localities in Du’an County and three localities in Dahua County, Guangxi, China. These five localities are in the assessment area of Southwest Karst National Park, which is currently being prepared. The extent of occurrence is about 960 km^2^ (< 5000 km^2^) and its occupancy area is predicted to continuously decline in the future due to grazing and firewood collection by local people. Therefore, according to the IUCN Red List Categories and Criteria (IUCN Standards and Petitions Committee 2022), *Tiliasaxatilis* should be considered in the Endangered (EN) [B1ab(iii)] category at present.

#### Additional specimens examined (paratypes).

China • Guangxi: Dahua County, Qibainong Town, Nonghe Village, Nongge, around the point 24.121001°N, 107.727671°E, limestone slope; alt. 880 m; 19 July 2023; *W. B. Xu, Z. C. Lu, M. L Mo, S. L. Chang & J. Q. Huang 16285* (IBK, KUN, PE) • ibid.; 19 July 2023; *W. B. Xu, Z. C. Lu, M. L Mo, S. L. Chang & J. Q. Huang 16312* (IBK, CSH, IBSC) • Du’an County, Bao’an Town, Shangzhen Village, Nongwen, around the point 24.07999417°N, 107.82876°E, limestone slope; alt. 769 m; 28 September 2023; *W. B. Xu, Z. C. Lu, M. L Mo, S. L. Chang & J. Q. Huang 17300* (IBK) • ibid.; 28 September 2023; *W. B. Xu, Z. C. Lu, M. L Mo, S. L. Chang & J. Q. Huang 17301* (IBK, GXMG, GXMI) • ibid.; 28 September 2023; *W. B. Xu, Z. C. Lu, M. L Mo, S. L. Chang & J. Q. Huang 17302* (IBK, GXMI) • ibid.; 28 September 2023; *W. B. Xu, Z. C. Lu, M. L Mo, S. L. Chang & J. Q. Huang 17303* (IBK, GXMG, CSH) • ibid.; 28 September 2023; *W. B. Xu, Z. C. Lu, M. L Mo, S. L. Chang & J. Q. Huang 17322* (IBK, PE, KUN) • Du’an County, Bao’an Town, Yuanli Village, Nongkou, around the point 24.10944°N, 107.811669°E, limestone slope; alt. 860 m; 30 September 2023; *W. B. Xu, Z. C. Lu, M. L Mo, S. L. Chang & J. Q. Huang 17367* (IBK) • ibid.; 30 September 2023; *W. B. Xu, Z. C. Lu, M. L Mo, S. L. Chang & J. Q. Huang 17368* (IBK) • Dahua County, Bansheng Town, Nongcong Village, Nongji, around the point 24°13′21.75″N, 107°45′25.99″E, limestone slope; alt. 936 m; 1 October 2023; *W. B. Xu, Z. C. Lu, M. L Mo, S. L. Chang & J. Q. Huang 17403* (IBK) • Dahua County, Qibainong Town, Nongjing Village, Baxiang, around the point 24.088763°N, 107.773282°E, limestone peak; alt. 920 m; 4 October 2023; *W. B. Xu, Z. C. Lu, M. L Mo, S. L. Chang & J. Q. Huang 17503* (IBK).

#### Notes.

*Tiliasaxatilis* has glabrous branchlets, leaf blades base oblique, truncate or cordate, abaxially hairy only in vein axils, bracts narrowly oblong, adnate to inflorescence peduncle, sessile, staminodes 5 and fruits indehiscent. Based on these morphological characters, *T.saxatilis* is similar to *T.tuan* (Tang et al. 2007), but differs from the latter in having leaf blades that are oblong or ovate-oblong, margin entire, fruit ellipsoid, 5-angled, apex acute. The morphological differences between *T.saxatilis* and *T.tuan* are shown in Table 1.

**Table 1.** Morphological characters distinguishing *T.saxatilis* from *T.tuan*.

Characters	*T.saxatilis*	*T.tuan*
Branchlets	Glabrous	Glabrous or tomentose
Leaf blades	Oblong or ovate-oblong, margin entire	Narrowly ovate or ovate-oblong to ovate-orbicular, margin entire or with a few minute teeth near apex or prominently dentate
Cymes	5–16-flowered, 3.5–6 cm long	3–22-flowered, 5–14 cm long
Bracts	3.8–9 cm long, 1–2 cm wide, sessile	6–16 cm long, 1–3 cm wide, sessile or with stalks 5–8 mm long
Pedicel	2–4 mm long	4–9 mm long
Fruit	Ellipsoid, 5-angled, apex acute	Globose or obovoid-globose, not ridged, apex rounded

In addition, several schemes for subdividing the genus *Tilia* have been described (Chang 1989; Zhuge and Tang 1995; Pigott 2012) and, according to the different morphological characteristics, *T.saxatilis* would be placed in different position. Chang (1989) gave two sections for *Tilia* in the "Flora Republicae Popularis Sinicae"; *T.saxatilis* would be placed in the sect. Tilia L. based on the fruit indehiscent when dry. Zhuge and Tang (1995) divided *Tilia* into three sections; *T.saxatilis* would be placed in the sect. Lindnera Reichb. based on the woody fruit exocarp. In Pigott’s (2012) monograph, the genus *Tilia* was divided into four sections; *T.saxatilis* would be placed in the section Astrophilyra V. Engler based on the leaf blades upper surface green, glabrous, lower surface pale green, hairy only in vein axils, fruits not splitting, flowers with staminodes.

## Supplementary Material

XML Treatment for Tilia saxatilis

## ﻿Additional information

### Author ORCIDs

Zhao-Cen Lu https://orcid.org/0000-0002-1725-0247

Shi-Li Chang https://orcid.org/0009-0005-9801-4121

Ming-Lin Mo https://orcid.org/0009-0000-8895-5474

You-Dong Wu https://orcid.org/0009-0006-6650-027X

Wei‑Bin Xu https://orcid.org/0000-0002-5602-8753

### Data availability

All of the data that support the findings of this study are available in the main text.
